# Evaluation of ViroCyt^®^ Virus Counter for Rapid Filovirus Quantitation

**DOI:** 10.3390/v7030857

**Published:** 2015-02-20

**Authors:** Cynthia A. Rossi, Brian J. Kearney, Scott P. Olschner, Priscilla L. Williams, Camenzind G. Robinson, Megan L. Heinrich, Ashley M. Zovanyi, Michael F. Ingram, David A. Norwood, Randal J. Schoepp

**Affiliations:** 1 Diagnostic Systems Division, US Army Medical Research Institute of Infectious Diseases, Fort Detrick, MD 21702, USA; E-Mails: brian.j.kearney.civ@mail.mil (B.J.K.); scott.p.olschner.civ@mail.mil (S.P.O.); priscilla.l.williams4.ctr@mail.mil (P.L.W.); mheinrich7295@gmail.com (M.L.H.); azovanyi@gmail.com (A.M.Z.); michael.f.ingram.mil@mail.mil (M.F.I.); david.a.norwood.civ@mail.mil (D.A.N.); randal.j.schoepp.civ@mail.mil (R.J.S.); 2 Pathology Division, US Army Medical Research Institute of Infectious Diseases, Fort Detrick, MD 21702, USA; E-Mail: robinsonc@janelia.hhmi.org

**Keywords:** ViroCyt^®^ Virus Counter, filovirus, quantitation, qRT-PCR, TEM, plaque assay

## Abstract

Development and evaluation of medical countermeasures for diagnostics, vaccines, and therapeutics requires production of standardized, reproducible, and well characterized virus preparations. For filoviruses this includes plaque assay for quantitation of infectious virus, transmission electron microscopy (TEM) for morphology and quantitation of virus particles, and real-time reverse transcription PCR for quantitation of viral RNA (qRT-PCR). The ViroCyt^®^ Virus Counter (VC) 2100 (ViroCyt, Boulder, CO, USA) is a flow-based instrument capable of quantifying virus particles in solution. Using a proprietary combination of fluorescent dyes that stain both nucleic acid and protein in a single 30 min step, rapid, reproducible, and cost-effective quantification of filovirus particles was demonstrated. Using a seed stock of Ebola virus variant Kikwit, the linear range of the instrument was determined to be 2.8E+06 to 1.0E+09 virus particles per mL with coefficient of variation ranging from 9.4% to 31.5% for samples tested in triplicate. VC particle counts for various filovirus stocks were within one log of TEM particle counts. A linear relationship was established between the plaque assay, qRT-PCR, and the VC. VC results significantly correlated with both plaque assay and qRT-PCR. These results demonstrated that the VC is an easy, fast, and consistent method to quantify filoviruses in stock preparations.

## 1. Introduction

Filoviruses are the causative agents of severe hemorrhagic fever with high mortality rates in humans [1]. They are enveloped, single-stranded, negative-sense RNA viruses belonging to two genera, *Ebolavirus* and *Marburgvirus*. There are at least five *Ebolavirus* species and a single *Marburgvirus* species [2]. Filovirus outbreaks are sporadic, rendering development and evaluation of therapeutic and vaccine efficacy in human trials problematic. For these virulent pathogens, the United States Food and Drug Administration (FDA) has established guidelines for the use of animal models as surrogates for human efficacy for the purpose of licensure. Well-characterized low passage virus preparations are critical in the development and evaluation of all medical countermeasures for these and other potential human pathogens. For this reason, standardized lots of filoviruses are required that are reproducible, highly characterized, and monitored over time for changes in the properties that could adversely affect its use as challenge stock in therapeutic or vaccine studies. The material produced must be of the highest quality, preferably with the highest infectious concentration (titer) and the lowest genomic equivalents (viral genetic material) to infectious virus and the lowest particle to infectious virus ratios achievable. Low ratios are thought to represent an intact virus and fewer numbers of non-infectious or defective interfering particles [3,4]. One of the keys to success is accurate virus quantification.

The most commonly used methods for filovirus quantification include the plaque assay and transmission electron microscopy (TEM). The gold standard for quantifying infectious viruses is the plaque assay, which provides a titer based on virus-infected cells producing a plaque. In theory, each plaque arises from a single infectious virion. Results provide a concentration of infectious virus particles, termed plaque-forming units (PFU/mL) [5]. Plaque assays are dependent on living cells and are labor intensive, with filoviruses requiring more than 7 days in culture and the requirement for high-level biocontainment (biosafety level-4). Generating consistent, reproducible viral titers using the plaque assay is dependent on a number of key parameters: cell type, cell confluency, inoculum volumes, agarose concentrations of primary and secondary overlays, cell staining reagent and concentration, as well as the day cells are stained and plaques are counted. While the tissue culture infectious dose 50% assay can also be used to quantitate infectious virus, it is not commonly used by the filovirus community. TEM has been used for many years to determine the number of virus particles and establish the shape and size of imaged particles on a grid. This technique does not measure infectivity or viability of the virus particles. Results are expressed as virus particles per mL (VP/mL). TEM is expensive, and sample preparation in high-level containment is challenging, tedious, and requires a skilled technician. Preparation and staining of grids takes a minimum of 2 h. Filovirus particle size is fairly well understood with length varying up to 14,000 nm and diameters of about 98 nm. Various configurations have been noted to include filaments, circular, and ‘6’-shaped forms. Counting requires a microscopist and it can take upwards of 2 h to count a single grid.

Quantitative reverse transcription-polymerase chain reaction (qRT-PCR) is a relatively new technique that enumerates genetic material by comparing an individual sample to a standard RNA curve. Results are expressed as genomic equivalents per mL (GE/mL). While the genomic sequences of filoviruses are very similar, different species can be distinguished using primers and probes with unique sequences [6,7]. Strain-specific quantitative real-time RT-PCR assays have been developed for use in our laboratories with EBOV primers targeting the glycoprotein, SUDV primers targeting the nucleoprotein, and MARV primers targeting the matrix protein, VP40 [8]. After inactivation by addition of TRIzol LS to the sample, all subsequent procedures can be completed outside of biocontainment.

The ViroCyt^®^ Virus Counter (VC) 2100 (ViroCyt, Boulder, CO, USA) instrument is a reengineered non-sorting analytical flow cytometer developed specifically to directly quantify virus particles in solution independent of the virus species. A single universal staining step uses a proprietary stain that is a combination of two fluorescent dyes, one specific for envelope proteins and the other specific for nucleic acids (both DNA and RNA, double stranded and single stranded) [9,10]. Virus particles containing both envelope proteins and nucleic acids are stained by both dyes. Stained proteins larger than 25 nm and a genome > 8kb are enumerated separately. As the stained virus passes through the laser the VC simultaneously detects both fluorescent signatures and is counted as an intact virus (particle). Unlike traditional flow cytometry, which uses a forward scatter trigger based on size as a criterion, the VC uses a fluorescent trigger to register an event. A minimum of 200 fluors per virus are required to accurately trigger counting events on the VC. Threshold values automatically determined during sample acquisition and data analysis are used to discriminate virus events from background signal. In addition, in order to eliminate the possibility of calculating a particle concentration based on a less than significant number of events (5E+05 VP/mL), the minimum number of simultaneous events must be at least 600. Samples containing large amounts of protein can elevate the background intensity. The purer or cleaner the sample matrix is, the easier it is to differentiate virus from background. Accurate results require that background values be as low as possible in order to capture as many events as possible while at the same time excluding background noise. When testing a new or unknown sample matrix, a series of dilutions should be tested to determine the best dilution to use in subsequent analyses. Previous testing of various dilutions of filovirus seed stocks revealed that this sample matrix must be diluted at least 1:10 in order to differentiate virus particles from background. Optimal dilution in this matrix was determined to be 1:25. The staining process for the VC takes 30 min and does not require a wash step. Total analysis time is less than 10 min per sample (in triplicate). Training required to operate the VC is minimal and data analysis takes place in real time. Preparation and staining of samples and use of the VC for filoviruses must occur in high containment. Use of the instrument also poses safety concerns, which must be addressed appropriately. Due to its design there is an unlikely but potential risk for aerosol generation at the waste end. To mitigate this risk, a HEPA (High Efficiency Particulate Air) filter can be added to the outlet valve on the waste bottle. The VC can be placed and operated inside a standard biosafety cabinet. A large number of viruses have been counted on the instrument, including several influenza strains, baculovirus, dengue type 1 virus, human coronavirus NL63, herpes simplex virus type 1, Rubella virus, Parainfluenza virus 2, and respiratory syncytial virus [10,11]. Like filoviruses, influenza viruses can present as filamentous particles and these can be easily counted by the instrument. The ease of sample preparation, speed of the assay, and less subjective interrogation of particles make this technique a potential addition to TEM for counting virus particles from filovirus stock preparations.

Infectious titer, genomic equivalents, and particle count are all quantitative measurements that must be accurate, reproducible, and robust for the results to be meaningful. Use of well characterized stocks in pivotal efficacy studies requires these assays to be standardized and validated. To evaluate the ViroCyt^®^ Virus Counter 2100 instrument and suitability for filovirus particle quantitation, we compared the VC to TEM, using a number of different filovirus stocks propagated in Vero E-6 (African green monkey kidney, Clone E-6) cells. In addition, we investigated the relationship between the different methods used to quantitate filoviruses, providing valuable insight into results derived from these various techniques.

## 2. Methods and Materials

### 2.1. Virus Preparations

All filovirus seed stocks used were media (supernatant) harvested from infected Vero E-6 cells after visual evidence of cytopathic effect in at least 50% of the cells. Media contained 10% fetal bovine serum. After harvesting, the medium was clarified, aliquoted into single use vials, and immediately stored at −70°C. TEM conducted shortly after harvest revealed morphology typical of filoviruses with virus particles present in numerous forms, predominately straight filamentous shapes, but torus, “6,” and “V” shapes were also present. Lengths ranged from 500 nm to excess of 2500 nm. Particle diameters were not reported.

A seed stock of Ebola virus (EBOV), Kikwit variant, was diluted beginning at 1:10 into Hank’s balanced salt solution buffer. Four serial ¼ log dilutions were prepared from the 1:10 in order to provide samples that lie within the linear range of the VC. Four additional serial 10-fold dilutions were prepared from the 1:10 dilution (1:100 to 1:1,000,000) for a total of nine dilutions (samples). The stock used to prepare diluted samples had an initial plaque assay titer of 1.78E+07 PFU/mL. The coded samples were tested with each quantitative method and used to examine reproducibility, linearity, and correlation between the various methods. Previously characterized stocks of EBOV, Kikwit variant, Sudan virus (SUDV), Gulu variant, and Marburg virus (MARV), Angola variant, were analyzed to compare the VC and TEM particle concentrations.

### 2.2. Plaque Assay

Each sample generated was diluted in a serial 10-fold series and tested in a viral plaque assay using confluent six-well plates of Vero E-6 cells, as described in Shurtleff* et al.*, 2012. Briefly, six wells were inoculated with 200 μL of each dilution and plates rocked every 15 min to minimize drying out of the monolayer. After 1 h incubation, 2 mL of a 0.5% semi-solid agarose overlay was added to each well. Plates were incubated for seven days and cells stained with 2 mL of a secondary overlay that included 5% neutral red (Gibco Life Technologies, Grand Island, NY, USA). Plaques in each well were counted 24 h post-staining. The titer for each sample was calculated from wells with countable plaques (≤150) and factoring in the dilution. Final concentration of the seed stock was established using results from all samples with plaque counts between 10 and 150.

### 2.3. Quantitative RT-PCR

Each sample was tested in triplicate with an EBOV-specific qRT-PCR assay developed and validated in our laboratory [8,12]. Briefly, synthetic viral RNA representative of the target region of the EBOV-Kikwit assay diluted in RNase free water was used to generate a standard curve for the determination of EBOV-Kikwit variant concentration. Samples were placed into TRIzol LS (Life Technologies, Carlsbad, CA, USA) and after removing from biocontainment, the RNA was purified using the QIAamp Viral RNA mini kit according to the manufacturer instructions (QIAGEN Inc, Valencia, CA, USA). Volumes pre- and post-extraction were equivalent. Control synthetic RNA and samples were assayed, in triplicate, on the ABI 7500 instrument (Applied Biosystems®, Life Technologies). Extracted samples were assayed undiluted. Synthetic RNA, quantified in genomic equivalents per reaction (GE/reaction), was run in serial 10-fold dilutions from 1E+09 to 1E+02 GE/reaction. ABI software used the synthetic RNA crossing threshold (Ct) values to calculate the slope, y-intercept, and *R*^2^ values for the standard curve. Using the Ct value for each sample, the GE/reaction was interpolated from the slope and y-intercept of the standard curve. The TRIzol dilution factor was then used to calculate the GE/mL for each sample. Final concentration of the seed stock was established using all sample results that fell within the linear range of the assay and factoring in the dilution of the sample.

### 2.4. Virus Particle Counts

#### 2.4.1. Transmission Electron Microscopy

An equal mixture of the sample (filoviral particles) and 1:100 dilution of 100 nm polystyrene beads (SPI Supplies, West Chester, PA, USA) were adsorbed to charged 200-mesh formvar/carbon-coated nickel grids (SPI Supplies), fixed using 2% cacodylate-buffered glutaraldehyde, and sterilized by exposure to vapors from 1% osmium tetroxide before being removed from biocontainment. Three grids were prepared for each sample. All viral particles and polystyrene beads were counted in at least 10 randomly chosen squares on each grid. The volume was calculated by dividing the number of beads counted by the known concentration per mL (2E+10). The bead solution: viral stock solution ratio was 1:1, so VP/mL was determined by dividing counted particles by the calculated volume of sample:

(1) $$V=\frac{beads\;counted}{bead\;concentration\;per\;ml}$$

(2) $$\text{Viral particles per mL}=\frac{viral\;particles\;counted}{V}$$

Final virus particle concentration of the seed stock was established using results from samples with countable number of particles on all three grids and factoring in the dilution of the sample.

#### 2.4.2. ViroCyt^®^ Virus Counter 2100 Instrument

The VC reagent kit (ViroCyt) was used following the manufacturer-recommended procedures. The instrument performance was validated prior to testing samples by running a non-biological positive control (stained beads performance validation standard used as an instrument performance check) and cleanliness control to verify the flow path was clean. Briefly, 300 μL of each sample was stained using 150 μL of Combo Dye solution, incubated in the dark at room temperature for 30 min, and analyzed in the VC. Samples were tested in triplicate with inter-sample washes and a cleanliness control run between each sample. Results were automatically analyzed by the instrument software and reported as VP/mL. A negative control sample was diluted and tested to establish the sample quantification limit [13]. The negative control sample was a clarified medium collected from uninfected Vero E-6 cells, prepared in parallel with our EBOV preparation. All VC results greater than this value were considered statistically distinguishable from the negative control (background) and therefore reported. The sample quantification limit was determined using the following equation:

(3) $$\text{Sample quantification limit}={\text{X}}_{\text{neg}}+{\text{t}}_{\text{99\%}}({\text{σ}}_{\text{neg}})$$

X_neg_ is the mean value of the negative control samples; σ_neg_ is the standard deviation of the negative control samples; and t_99%_ is the t value for N-1 degrees of freedom at the 99% confidence level.

Final virus particle concentration of the seed stock was established using all samples whose VP/mL counts were above the sample quantification limit and within the linear range of the instrument and factoring in the dilution of the sample.

### 2.5. Statistical Analysis

Microsoft Excel 2007 (Redmond, WA, USA) was used for linear-regression analysis. GraphPad Prism v5 software (LaJolla, CA, USA) was used to perform Pearson correlation analysis to determine the correlation between each assay (correlation coefficients (r) and *p*-values). A *p*-value of <0.05 was considered statistically significant.

## 3. Results

**Figure 1:**
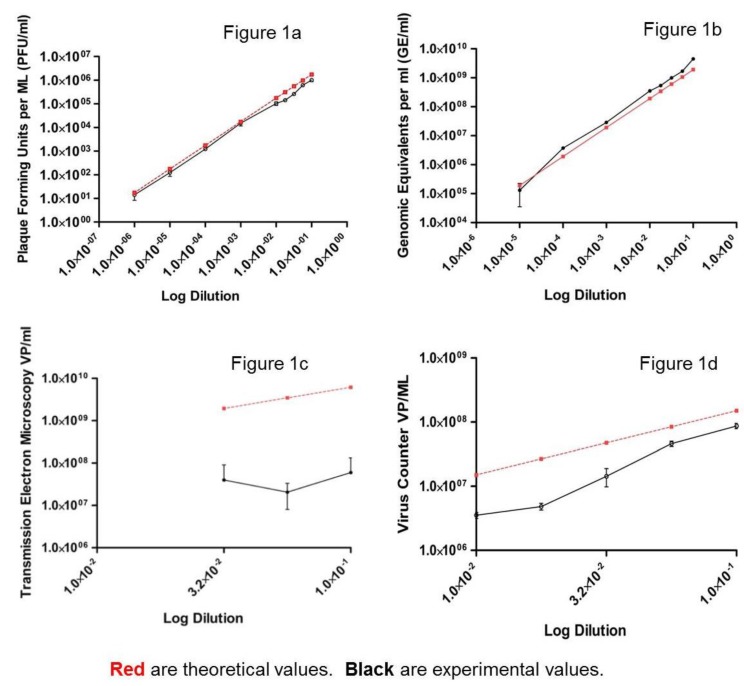
Log-log plot of dilution* versus* concentration for a series of Ebola virus (EBOV), Kikwit variant, samples produced by diluting the stock virus. Theoretical results are shown in red and are based on the original stock concentration determined at time of preparation. Experimental results are shown in black. (**a**) Infectious virus was quantified for each sample using six well plates of confluent Vero E-6 cells and an agarose-based plaque assay (PFU/mL). All samples were tested in six replicates; bars represent standard deviation. (**b**) Genomic equivalents were quantified for each sample using an EBOV-specific qRT-PCR (GE/mL). All samples were tested in triplicate; bars represent standard deviation. (**c**) Virus particle concentration (VP/mL) was quantified from each sample using transmission electron microscopy (TEM). All samples were tested in triplicate. Bars represent standard deviation. (**d**) Virus particle concentration was quantified for each sample using the ViroCyt^®^ Virus Counter 2100 instrument. All samples were tested in triplicate; bars represent standard deviation.

A total of nine dilutions (samples) were generated using an EBOV, Kikwit variant stock. Samples were coded and then tested using the various quantitative methods. Results are shown in Figure 1.

Every sample was quantified by plaque assay (Figure 1a). The number of plaques used to determine each sample’s titer were between 18 and 58 per well except for the most dilute sample (1:1,000,000 dilution), which had between 1 and 4 plaques per well. Using all nine samples, the *R*^2^ for the experimental plaque assay result was 0.9984 with a slope of 0.9633. Experimental titers correlated well to theoretical values (Pearson *r* = 0.9967, *p* value < 0.0001). Using the 8 samples whose titers were established using plaque counts between 10 and 150, the concentration of the stock virus was determined to be 1.10E+07 PFU/mL, a difference of 0.21 logs from the original titer (1.78E+07 PFU/mL). The coefficient of variation (CV) for the first seven samples ranged from 7.4% to 22.3%, with 27.8% and 37.7% for the two most dilute samples.

The EBOV-specific qRT-PCR assay was linear between 1E+02 and 1E+09 GE of synthetic RNA per reaction (Figure 1b). *R*^2^ for the standard curve used to determine GE/mL was 0.999 with a slope of −3.48 and an intercept of 45.08 (data not shown). The lowest copy number quantified by the qRT-PCR corresponded to a 1:10,000 dilution of the stock virus (equivalent to 1.23E+03 PFU/mL). While detected, results from the 1:100,000 dilution fell outside the linear range of the assay (CV = 72.9%). Using seven samples whose results fell within the linear range of the assay, the *R*^2^ for the experimental results was 0.9961 with a slope of 1.006. Experimental titers correlated well to theoretical values (Pearson *r* = 0.9824, *p* value < 0.0001). The GE/mL of this seed stock was determined to be 3.40E+10, a difference of 0.25 logs from the original GE/mL concentration (1.91E+10 GE/mL). CV ranged from 1.2% to 7.4% for those samples that fell within the linear range of the assay.

Three grids of each dilution (sample) were prepared and counted using TEM. Particle morphology was not included in this assessment. Virus particles were not detected in sample dilutions greater than 1:31.6 (equivalent to 2.63E+05 PFU/mL). Results from the TEM are shown in Figure 1c. In the most concentrated sample, virus particles from all three grids were counted and yielded a particle concentration, while only two of three grids for the other two samples had virus particles. Using all three samples, the *R*^2^ for the experimental results was 0.1438 with a slope of 0.36. Experimental titers did not correlate with theoretical values (Pearson *r* = 0.6783, *p* value = 0.5254). Using only the most concentrated sample, the particle concentration was determined to be 4.01E+07 VP/mL for this stock, a difference of 0.82 logs from the original VP/mL concentration (2.67E+08 VP/mL). The CV for replicate samples was 121.6%.

Uninfected Vero E-6 cell culture media was used to determine the background or sample quantification limit of the VC. Using a total of 15 values, we determined the sample quantification limit of the instrument to be 2.8E+06 VP/mL (data not shown). Sample VP/mL results equal to or below this value were reported as below the limit of detection of the instrument (*i.e.*, the instrument qualification limit). Three replicates of each dilution (sample) were tested in the VC. No virus particles were detected in sample dilutions greater than 1:100 (equivalent to 1.03E+05 PFU/mL). Results from the VC are shown in Figure 1d. Using five samples with concentrations in the linear range of the instrument (2.8E+06 to 1.0E+09 VP/mL), the *R*^2^ value for the experimental results was 0.9731 with a slope of 1.5045. Experimental titers correlated well to theoretical values (Pearson *r* = 0.9940, *p* value = 0.0006). Particle concentration was determined to be 5.5E+08 VP/mL for this stock, a difference of 0.44 logs from the original VP/mL concentration (1.5E+09 VP/mL). CV ranged from 9.4% to 31.5% for those samples that fell within the linear range of the instrument. Similar dilution analyses were done for a number of filoviruses using the VC, with all stocks yielding linear regression fits of *R*^2^ ≥ 0.95 and slopes between 1.24 and 1.58. The *R*^2^ values and slopes confirm a strong positive correlation and linear relationship between VC results and dilution values (data not shown).

**Figure 2:**
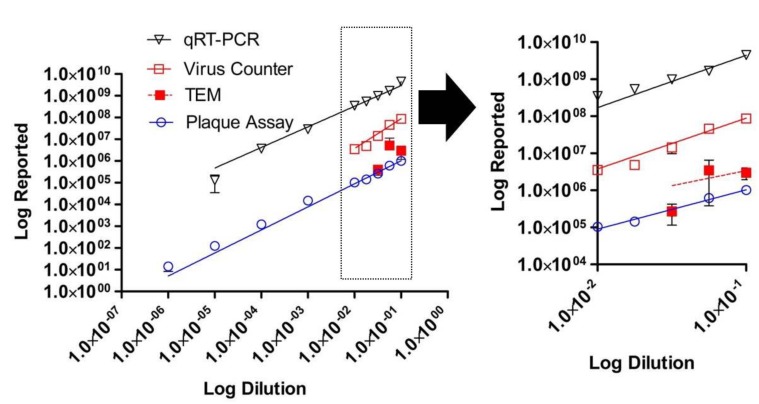
Log-log plot of dilution* versus* reported concentration for a series of Ebola virus (EBOV), Kikwit variant, samples produced by diluting the stock virus. Infectious virus was quantified for each sample using six well plates of confluent Vero E-6 cells and an agarose-based plaque assay (PFU/mL). All samples were tested in six replicates; bars represent standard deviation. Genomic equivalents were quantified for each sample using an EBOV-specific qRT-PCR (GE/mL). All samples were tested in triplicate; bars represent standard deviation. Virus particles concentration (VP/mL) was quantified from each sample using transmission electron microscopy (TEM). All samples were tested in triplicate. Bars represent standard deviation. Virus particle concentration was also quantified for each sample using the ViroCyt^®^ Virus Counter 2100 instrument. All samples were tested in triplicate; bars represent standard deviation.

**Figure 3:**
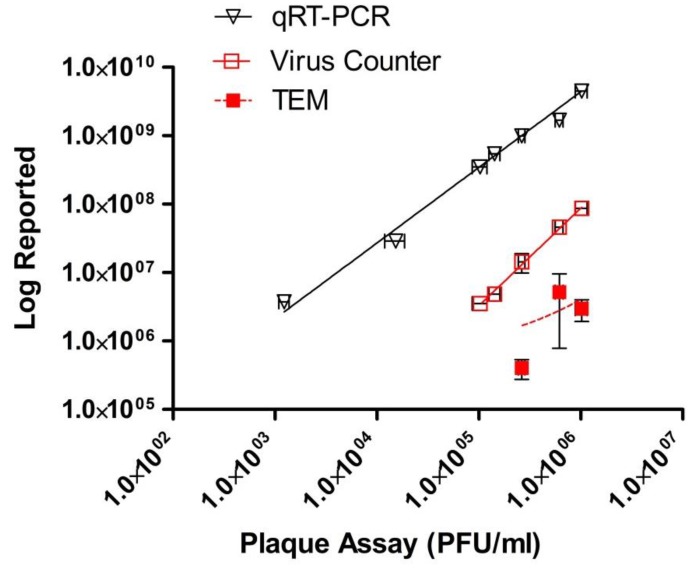
Log-log plot for a dilution series of Ebola virus (EBOV), Kikwit variant, stock virus. Infectious virus was quantified for each sample using six well plates of confluent Vero E-6 cells and an agarose-based plaque assay (PFU/mL) (six replicates) and compared to nucleic acid concentration and particle counts. Nucleic acid concentration, reported as genomic equivalents per mL, was determined using an EBOV-specific qRT-PCR (three replicates). Virus particle counts per mL were measured using the ViroCyt^®^ Virus Counter 2100 instrument and transmission electron microscopy (TEM) (three replicates each). Bars represent standard deviation.

**Figure 4:**
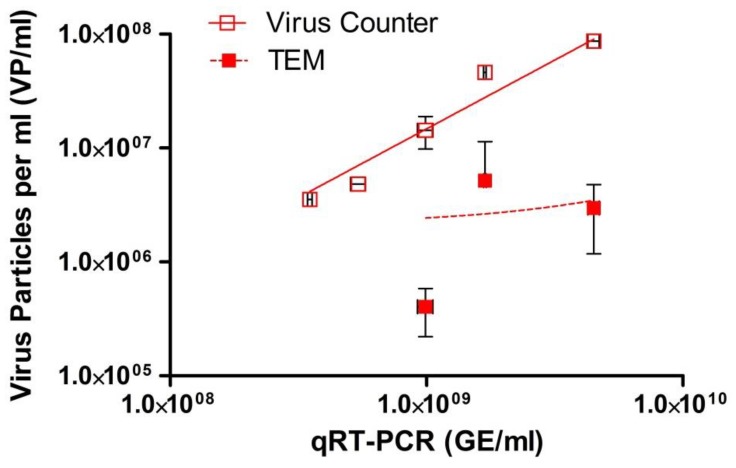
Log-log plot for a dilution series of Ebola virus (EBOV), Kikwit variant, stock virus. Reported nucleic acid concentration (genomic equivalents per mL) for each sample as measured by a EBOV-specific qRT-PCR was compared to the reported virus particle counts per mL measured using the ViroCyt^®^ Virus Counter 2100 instrument and transmission electron microscopy (TEM). All samples were tested in triplicate; bars represent standard deviation.

The relationship between infectious virus, genomic material, and virus particles as measured by VC and TEM are shown in Figure 2. These results demonstrate that there is a linear relationship for each assay with *R*^2^ values > 0.97 for all methods except TEM (0.14) and slopes of 0.96 for the plaque assay, 1.01 for the qRT-PCR, 1.50 for the VC, and 0.36 for TEM. As presented in Figure 3, our study demonstrates that the VC and qRT-PCR quantitative techniques correlated well with the traditional plaque assay; however, the TEM particle count did not. Figure 4 demonstrates that only the VC particle counting technique correlated with the EBOV-specific qRT-PCR. The TEM particle count did not correlate. There is no correlation between the two virus particle quantitative methods when using TEM results for all three dilutions (*p* = 0.4649) (data not shown).

A summary of the initial quantitative assay results for the EBOV, Kikwit variant stock described above is shown in Table 1 (Prep 1). Additional quantitative assay results for a number of different filovirus stocks are also shown in Table 1. In general, particle counts were greater than those of the plaque assay, but lower than those generated from the qRT-PCR. The differences between the two particle counting methods are shown for each virus stock and range from 0.23 to 0.91 logs.

**Table:** 

	Pearson correlation			Linear regression	
Plaque assay compared to:	*r* =	2-tailed *p* =	Significant (alpha = 0.05)	*R^2^* =	Slope
qRT-PCR	0.9755	0.0002	Yes	0.903	1.06
TEM	0.7633	ns	No	0.1768	0.45
Virus Counter	0.9984	<0.0001	Yes	0.9965	1.44

**Table:** 

	Pearson correlation			Linear regression	
qRT-PCR compared to	*r* =	2-tailed *p* =	Significant (alpha = 0.05)	*R^2^* =	Slope
TEM	0.5414	ns	No	0.4661	0.66
Virus Counter	0.9777	0.0040	Yes	0.9576	1.36

**Table 1:** Quantitative assay results for different filovirus stocks; Ebola virus (EBOV), Sudan virus (SUDV), and Marburg virus (MARV). Infectious virus titer for each stock as determined by agarose-based plaque assay (PFU/mL), nucleic acid concentration reported as genomic equivalents per mL as measured using a virus-specific qRT-PCR (GE/mL), and virus particle counts per mL (VP/mL) measured using the ViroCyt^®^ Virus Counter 2100 instrument and transmission electron microscopy (TEM). The log difference in particle counts between the Virus Counter and TEM methods is shown in the last column.

Virus	Plaque assay (PFU/mL)	Virus-specific qRT-PCR (GE/mL)	Virus counter (VP/mL)	TEM (VP/mL)	Log difference between virus counter and TEM
EBOV, Kikwit variant, Prep 1	1.78E+07	1.91E+10	1.50E+09	2.67E+08	0.75
EBOV, Kikwit variant, Prep 2	4.56E+06	2.35E+10	7.50E+08	6.10E+09	0.91
EBOV, Kikwit variant, Prep 3	7.02E+06	2.33E+10	1.62E+09	4.63E+08	0.57
EBOV, Kikwit variant, Prep 4	1.31E+06	6.42E+09	5.55E+08	1.17E+09	0.32
SUDV, Gulu variant	9.10E+06	1.73E+10	7.13E+08	3.84E+08	0.27
MARV, Angola variant	2.88E+07	3.89E+10	1.03E+09	1.76E+09	0.23

## 4. Discussion

Current filovirus quantitation methods include plaque assay, qRT-PCR, and TEM. Virus quantitation-specific assays used by different laboratories vary depending on the size of the biocontainment space, user preferences, and specific needs. Filovirus plaque assays are the gold standard assay for quantitation. Plaque assay quantifies infectious virus and has been used for many years by filovirus researchers. The assay is biological in nature, depending on living cells and their ability to be infected and produce plaques. The assay measures infectious virus but the ratio of infectious virus particle to plaque is unknown. There are many steps that contribute to the accuracy and reproducibility of the results. Standardization and training with periodic assessment can ensure the results are always optimal. Repeatability, a component of precision, was defined in this study as the CV between replicates. The plaque assay CV ranged from 7.4% to 37.7%, with the most dilute samples exhibiting the highest values. Titers established for each of these samples were within 0.28 logs. Reproducibility, expressed in this study as the difference in titer between the stock concentration determined at the time of preparation and that achieved in this study, was very good with only a 0.21 log difference in titer (97.1% of the original titer).

Quantitative RT-PCR measures genetic material (RNA) that is specific for the primers and probes used in the assay. qRT-PCR has been used to quantify many viruses to include influenza [14], human immunodeficiency virus [15], and dengue [16]. Due to the amplification of nucleic acid, this assay is very sensitive and has a wide linear range, which facilitates quantitation. Designing primers and probes requires substantial expertise. Amplification occurs from both genomic as well as free nucleic acids as long as the target sequence is present in the sample [11,17]. The assay is relatively quick (1–2 h) but due to multiple steps and its sensitivity, the technique can be prone to error and contamination. It is relatively expensive, requiring a virus-specific set of primers and probes, a reproducible source of control material, and a skilled technician to minimize contamination. During the validation the lower limit of quantitation of the EBOV-specific qRT-PCR was shown to be 1E+02 GE per reaction. For this EBOV stock this was equivalent to 1.23E+03 PFU/mL. Repeatability of this assay was excellent with CVs between 1.2% and 7.4% and GE/mL values within 0.19 logs for samples that fell within the linear range of the assay. Reproducibility was within 0.25 logs of the original concentration (102.4%).

Virus particle count by TEM quantifies the number of virions meeting specific criteria of size and shape consistent with filoviruses. Filovirus particle size is fairly well established but problematic since the virus is pleomorphic with varying proportions of long filaments, ‘6’-shaped forms, and circular configurations. Although nucleocapsid and particle diameters were consistent within and among species, the average particle length was 996 nm but varied up to 20,000 nm depending on the virus and cells used to propagate [18]. Particles containing a nucleocapsid have a diameter roughly 96–98 nm while those not containing a nucleocapsid are 48–52 nm [19], providing some guidance for only counting intact and potentially infectious particles.

TEM is faster than plaque assay and, like the qRT-PCR, takes only hours to complete. No virus-specific reagents are required. However, it is relatively expensive and few laboratories have the required expertise and instruments. TEM particle counts, although the current method of choice for enumeration of virus particles, have a relatively high limit of detection of 1E+07 VP/mL, which contributed to our inability to quantitate virus beyond the first dilution [20]. Different methods of sample preparation can lower the limit of detection to 1E+05 VP/mL, but use of these methodologies requires purification through sucrose density gradients or concentration by high-speed pelleting followed by fixation and embedding in resin [20]. Use of these methodologies would alter the sample composition and not provide a particle count, as found in the stock virus. TEM particle counts are also highly variable, with reports in the literature of relative error as high as 40% and standard deviations of up to 0.5 logs [21,22]. Variability may arise during grid preparation with loss of specimen and/or reference beads during sample fixation and grid washing. Significant challenges to the precision (repeatability) and reproducibility of TEM are the pre-fixation drying, fixation artifacts, uneven sample distribution, clumping of the sample and/or reference beads, and the background that is caused by media salts and proteins present in the sample matrix. Preparation of quality grids is especially challenging in a biosafety level-4 environment where dexterity is extremely limited. Counting is subjective, but the effects can be reduced by standardization, cross-training, and periodic assessment. In our study, repeatability or CV of this assay was 121.6% for the most concentrated sample tested. Reproducibility was within 0.82 logs or 90.2% of the original concentration. TEM particle counts did not correlate with any of the other three quantitative assays, mainly due to the lack of samples that could be compared. Dilutions of EBOV chosen for this study were designed to provide more samples that would fall within the VC quantitative limits since it was assumed TEM had the lower sensitivity. The low number of particles counted per grid and high CV noted in the most concentrated sample indicates that it is at or near the limit of detection for this assay (1.02E+06 PFU/mL). In this study TEM results proved to be problematic, arising from the quality of the prepared grids and the higher sensitivity.

The VC is a rapid method, providing real-time results for multiple samples in less than 1 h. Cost per sample, run in triplicate, is approximately $15 ($4.75 per replicate). Training to use and care for the instrument is minimal, but use of the instrument for filoviruses requires high containment and poses aerosol hazards that must be carefully mitigated. The one-step assay, instrument, and software are easy to use and do not require a highly trained individual. Due to its universal stain, the assay can be used with a wide variety of viruses. The VC is not amenable to quantifying filovirus particles directly from samples with high levels of proteins that are found in clinical samples (whole blood, serum, plasma) or stock preparations with fetal bovine serum concentrations greater than 10%. In these sample types, an initial dilution is required to lower background levels. Analysis of these matrices is possible, but would require purification or some other method to remove exogenous protein. While we can easily conceptualize automated counting of homogeneous spherical viruses such as herpes simplex, baculovirus, and dengue [10], the VC has been shown to reproducibly enumerate influenza A and B, which can contain filamentous particles [10,11]. For these viruses, VC results were shown to have a linear relationship and correlate with plaque assay, qRT-PCR, TEM, and/or tissue culture infectious dose 50% assays. The ability to count heterogeneous particles is due to the VC triggering on two simultaneous fluorescent events, or signals, as a virus particle passes through the laser and not the size or shape of the target as in conventional flow cytometry. Analysis of media only (negative) controls was required to establish the sample quantitation limit of the assay for this matrix (2.8E+06 VP/mL). For this EBOV stock this was equivalent to 1.03E+05 PFU/mL. Repeatability of this assay was similar to the plaque assay with CV ranging from 9.4% to 31.5%. VP/mL values were within 0.51 logs for samples that fell within the linear range of the assay. Reproducibility was within 0.44 logs (95.3%).

We have demonstrated that there is a linear relationship between the plaque assay, qRT-PCR, and VC particle counts. If higher virus concentrations were tested, work described in other studies [21] suggests that the TEM assay would also have been linear. The data presented in this study show that the VC is capable of providing rapid filovirus quantitation from seed stocks with results that significantly correlate with the plaque assay (*r* = 0.9984, *p* < 0.05) and the qRT-PCR (*r* = 0.9777, *p* < 0.05).

Applicability of the VC to other filovirus stocks is illustrated in Table 1. Results from four different EBOV Kikwit preps as well as a single variant of SUDV and MARV are presented. While no direct correlation between concentrations of the various virus stocks were noted, the particle counts were greater than the plaque titers but lower than the genomic equivalents. Variability seen is possibly due to the time or amount of cell lysis when each preparation was harvested or the multiplicity of infection (MOI) used to produce the stocks, all of which are known to affect the infectious virus titer. Kikwit preps 1 and 2 represent similar Vero E-6 passage three stocks prepared at an MOI of 0.01, while prep 3 and 4 represent passage two stocks of the same EBOV variant. Prep 3 was produced at an MOI of 0.01 and prep 4, like the SUDV Gulu and MARV Angola variants, were produced at an MOI of 0.001. Results reveal that there is about a log surplus of viral genetic material produced when compared to the number of virus particles as measured by the VC and an additional two logs more particles than infectious virus. These results may be due to the amount of cell lysis at the time of harvest or perhaps inefficient filovirus virion packaging or both. Similar patterns were noted for filoviruses by Weidman and colleagues when comparing plaque assay, qRT-PCR, and a focus-forming assay using virus-specific antibodies for a variety of hemorrhagic fever viruses [17]. Their study demonstrated that some viruses, for example Lassa and yellow fever, were more efficient at replication and packaging of infectious viruses than others such as filoviruses and Rift Valley fever virus. As shown in Table 1, the VC and TEM particle counts were within one log of each other, which is also within the normal variability seen for the TEM (data not shown). As TEM improvements are instituted and variability of this technique is improved this difference should decrease.

The VC assay is simple to conduct, training is minimal, and no virus-specific reagents are required. Due to the use of an instrument for analysis, quantification is non-subjective and requires <10 min per reaction. The instrument is relatively small and can be placed inside a standard biosafety cabinet; installation and operational qualifications have been established; and assay controls are available to verify the instrument is functional. While we do not claim that particle counts are substitutes for techniques that measure infectious virus or genomic material, we do feel that the use of the VC to quantitate virus particles in seed stocks is a viable alternative to the more challenging, less sensitive TEM method. At a minimum, the VC provides an additional tool for rapid, precise, reproducible, and cost-effective quantification of filovirus particles in stock preparations and could prove useful to monitor various steps during virus propagation or purification.
